# Testing differences statistically with the Leiden ranking

**DOI:** 10.1007/s11192-012-0636-6

**Published:** 2012-01-26

**Authors:** Loet Leydesdorff, Lutz Bornmann

**Affiliations:** 1 Amsterdam School of Communication Research, University of Amsterdam, Kloveniersburgwal 48, 1012 CX Amsterdam, The Netherlands; 2 Max Planck Society, Administrative Headquarters, Hofgartenstr. 8, 80539 Munich, Germany

**Keywords:** Ranking, University, Test, Comparison, Expectation

## Abstract

The Leiden ranking 2011/2012 provides the *Proportion top*-*10% publications* (*PP*_*top-10%*_) as a new indicator. This indicator allows for testing performance differences between two universities for statistical significance.

On 1 December 2011, the Centre for Science and Technology Studies (CWTS) at Leiden University launched the Leiden ranking 2011/2012 at http://www.leidenranking.com/ranking.aspx. The Leiden ranking 2011/2012 measures the scientific performance of 500 major universities worldwide. The *PP*_*top-10%*_ is added as a new indicator of impact. This indicator corresponds with the excellence indicator (*EI*) recently introduced in the SCImago Institutions rankings (at http://www.scimagoir.com/pdf/sir_2011_world_report.pdf).

Whereas SCImago uses Scopus data, the Leiden ranking is based on the Web-of-Science data of Thomson Reuters. In addition to the “stability intervals” provided by CWTS, values for both *PP*_*top-10%*_ and *EI* can be tested statistically for significant differences from expectation. Furthermore, the statistical significance of performance differences between universities can be tested by using the *z*-test for independent proportions (Bornmann et al. in press; Sheskin 2011, pp. 656f).

An Excel sheet can be downloaded from http://www.leydesdorff.net/leiden11/leiden11.xls into which the values for this indicator *PP*_*top-10%*_ can be fed in order to obtain a *z* value. The example in the download shows the results for Leiden University when compared with the University of Amsterdam (not statistically significantly different; *p* > 0.05), and for Leiden University when compared with the expectation (the value is statistically significant above the expectation; *p* < 0.001). The values in the sheet can be replaced with values in the ranking for any university or any set of two universities.

## The *z*-test

The *z*-test can be used to measure the extent to which an observed proportion differs significantly from expectation, and whether the proportions for two institutions are significantly different. In general, the test statistics can be formulated as follows:

$$ z = \frac{{p_{1} - p{}_{2}}}{{\sqrt {p(1 - p)\left[ {\frac{1}{{n_{1} }} + \frac{1}{{n_{2} }}} \right]} }} $$

where *n*_*1*_ and *n*_*2*_ are the numbers of all papers published by institutions 1 and 2 (under the column “*P*” in the Leiden ranking); and *p*_*1*_ and *p*_*2*_ are the values of *PP*_*top-10%*_ of institutions 1 and 2. The pooled estimate for proportion (*p*) is defined as:

$$ p = \frac{{t_{1} + t_{2} }}{{n_{1} + n_{2} }} $$

where *t*_*1*_ and *t*_*2*_ are the numbers of top-10% papers of institutions 1 and 2. These numbers are calculated (in the sheet) on the basis of “*P*” and “*PP*_*top-10%*_” provided by the Leiden ranking. When testing observed versus expected values for a single sample, *n*_1_ = *n*_2_. In that case, *p*_1_ is the value of the *PP*_*top-10%*_, *p*_2_ = 0.1, and *t*_2_ = 0.1 × *n*_2_ (that is, the expected number in the top-10%).

An absolute value of *z* larger than 1.96 indicates the statistical significance of the difference between two ratings at the 5% level (*p* < 0.05); the critical value for a test at the 1% level (*p* < 0.01) is 2.576. However, in a series of tests for many institutions, a significance level higher than 5% must be chosen because of the possibility of a family-wise accumulation of type-I errors (the so-called Bonferroni correction; cf. Leydesdorff et al. 2011).

In summary, it seems fortunate to us that two major teams in our field (Granada and Leiden University) have agreed on using an indicator for the Scopus and WoS databases, respectively, that allows for testing of statistically significant differences of scientific performances. Of course, there remains the problem of interdisciplinarity/multidisciplinarity when institutional units, such as universities are ranked. This could be counteracted by field-normalization and perhaps by fractionation of citations (1/the number of references) in terms of the citing papers (Zhou and Leydesdorff 2011).
